# Belarusian Healthcare Professionals’ Views on Monkeypox and Vaccine Hesitancy

**DOI:** 10.3390/vaccines11081368

**Published:** 2023-08-15

**Authors:** Abanoub Riad, Nadzeya Rybakova, Nadzeya Dubatouka, Ina Zankevich, Miloslav Klugar, Michal Koščík, Anton Drobov

**Affiliations:** 1 Department of Public Health, Faculty of Medicine, Masaryk University, 625 00 Brno, Czech Republic; koscik@med.muni.cz (M.K.); anton.drobov@med.muni.cz (A.D.); 2 Institute of Health Information and Statistics of the Czech Republic (IHIS-CR), 128 01 Prague, Czech Republic; klugar@med.muni.cz; 3 Czech National Centre for Evidence-Based Healthcare and Knowledge, Faculty of Medicine, Masaryk University, 625 00 Brno, Czech Republic; 4 Medical Center of Modern Pediatrics, Chervyakova Str. 55, 220053 Minsk, Belarus; nadina77543@gmail.com (N.R.); care@pediatrics.by (N.D.); 5 Bausch Health LLC, Olshevskogo Str. 22, 220073 Minsk, Belarus; nsp159@mail.ru

**Keywords:** cross-sectional studies, disease outbreaks, health belief model, health personnel, knowledge, monkeypox, Republic of Belarus, smallpox vaccine, vaccination hesitancy

## Abstract

Background: Despite the low transmission risk of Monkeypox (mpox) in Belarus, this study is vital as it contributes to our understanding of vaccine hesitancy among healthcare professionals (HCPs). It aims to assess vaccination perceptions and evaluate the willingness to pay for the vaccine among Belarusian HCPs, thereby enhancing pandemic preparedness. Methods: in October 2022, a cross-sectional survey-based study was conducted among Belarusian HCPs using a self-administered questionnaire (SAQ). Invitations were disseminated via social media platforms using a snowball sampling method. The SAQ encompassed various categories, including sociodemographic details, medical history, sources of mpox information, perceived and factual mpox knowledge, and perceptions of the mpox vaccine according to the health belief model (HBM), mpox vaccine acceptance and willingness to pay (WTP). Results: while a large proportion of respondents had good knowledge of mpox epidemiology and its clinical manifestations, their awareness of available vaccines and treatment options was limited. Consequently, a significant correlation was found between the history of influenza vaccination and mpox-related knowledge. Furthermore, the study showed that just over half of the participants (51.4%) were willing to receive the mpox vaccine if offered for free, safely, and effectively, with their decision largely influenced by perceived benefits (*Spearman’s rho* = 0.451) and cues to action (*Spearman’s rho* = 0.349). However, a considerable degree of hesitancy (30.6%) and resistance (18.1%) towards the mpox vaccine was observed, underscoring the need for targeted interventions to address these issues. Conclusions: this study highlights a significant knowledge gap among Belarusian HCPs about mpox vaccines and treatments, despite a general awareness of the disease’s epidemiology and symptoms, and it underscores the need for targeted interventions to enhance mpox knowledge and vaccine acceptance.

## 1. Introduction

Monkeypox (mpox) is a zoonotic orthopoxvirus that sometimes causes the disease in a human population with symptoms similar to smallpox, although with notably lower mortality [1]. The monkeypox virus (MPXV) was first identified and isolated when monkeys transported from Singapore to Denmark for research purposes contracted a vesicular disease. The name ‘monkeypox’ originated from this time [2]. The first human case was discovered in 1970 when the virus was isolated from a 9-year-old child in a rural area of the Democratic Republic of Congo (DRC) suspected of having smallpox [3].

From May 2022, multiple cases of mpox were confirmed worldwide in non-endemic countries [4]. Subsequently, the World Health Organisation (WHO) declared the global monkeypox outbreak as a public health emergency of international concern on 23 July 2022 [5,6]. The ongoing epidemic is primarily driven by human-to-human transmission through respiratory droplets, fomites, and direct contact with lesions of an infected individual [7]. Such transmission can easily occur in healthcare settings, putting healthcare workers at risk of contracting the disease. As of 23 May 2023, 87,545 laboratory-confirmed mpox cases have been identified worldwide. There were no reported cases in Belarus [8]. However, mpox cases were reported from all the neighbouring countries, such as Poland (217 cases), Latvia (6), Ukraine (5), Lithuania (5), and Russia (2). There were no fatal cases reported in the bordering countries [8]. As of 3 March 2023, only Lithuania has not started mpox vaccination of the neighbouring countries, while for Russia, the information is not available. There is no access to vaccination against mpox in Belarus yet [9]. Due to the lack of direct flight connections with most countries in the world, the transmission risk is considered low. According to the Ministry of Health of Belarus, measures of quarantine control have been strengthened at the border entry points [9]. Even though the risk of imported cases is low, the healthcare workers who first deal with such cases should be aware of the main measures of prevention and diagnostic features of novel emerging diseases, such as mpox.

Furthermore, knowledge gaps were identified among healthcare workers regarding clinical presentation and vaccination during a study in Saudi Arabia [10]. Another study conducted among Italian adults revealed significant knowledge gaps about the disease, its transmission, and prevention, emphasizing the need for effective communication and education strategies [11]. A recent systematic review by Lounis and Riad, 2023, found that in the early months of the mpox emergence, there was an unsatisfactory level of knowledge and awareness of the disease, particularly among healthcare professionals (HCPs) in non-endemic countries [12]. The review also highlighted a prevalent hesitancy towards the mpox vaccine, except for Chinese HCPs, who demonstrated a high acceptance rate of 90.1% [12]. 

According to WHO interim guidance on vaccines and immunisation for monkeypox updated on 24 August 2022, mass vaccination is not recommended nor required for monkeypox at the moment [6]. Meanwhile, the WHO recommended that pre-exposure prophylaxis (PrEP) should be administered to high-risk groups, including (a) HCPs at high risk of exposure, (b) laboratory personnel working with orthopoxviruses, (c) clinical laboratory personnel performing diagnostic testing for mpox, and (d) outbreak response team members (as designated by national public health authorities) [6].

The Health Belief Model (HBM) is a theoretical model that has been widely used to predict health behaviours and health service utilisation based on individual beliefs [13,14]. It postulates that health-related action depends upon the simultaneous occurrence of three classes of factors: (1) the existence of sufficient motivation (or health concern) to make health issues salient or relevant, (2) the belief that one is susceptible (perceived susceptibility) to a serious health problem or to the sequelae of that illness or condition, and (3) the belief that following a particular health recommendation would be beneficial in reducing the perceived threat, and at a subjectively acceptable cost (perceived benefits minus perceived barriers) [14,15]. In the context of our study, the HBM can help explain the choices or stated intentions of healthcare professionals regarding the mpox vaccine, as these choices are likely influenced by their perceived susceptibility to mpox, perceived benefits of the vaccine, and perceived barriers to vaccination.

Therefore, one of the primary goals of this study is to assess the levels of perceived knowledge and factual knowledge of HCPs, to evaluate perceptions of vaccination against monkeypox, and to assess levels of vaccine acceptance and willingness to pay (WTP). The findings from this study will inform targeted educational interventions and policy decisions regarding vaccination strategies, thereby enhancing the preparedness for potential monkeypox outbreaks.

## 2. Materials and Methods

### 2.1. Design

In accordance with the Strengthening the Reporting of Observational Studies in Epidemiology (STROBE) guidelines, a descriptive survey-based cross-sectional study was designed and conducted in October 2022 [16]. The study employed a self-administered questionnaire (SAQ), which was distributed and filled in electronically via the KoBoToolbox platform (Harvard Humanitarian Initiative; Cambridge, MA, USA, 2022) [17].

### 2.2. Participants

The focus of this study was on Belarusian healthcare professionals (HCPs) who may or may not have been engaged in offering clinical care to monkeypox (mpox) cases during the 2022 outbreak. Participants were required to meet the following inclusion criteria: (i) employment, either full-time or part-time, with a healthcare provider operating within Belarusian jurisdictions, and (ii) having responsibilities related to the provision of clinical services. Individuals were excluded from the study if they (i) held administrative, economic, or legal roles within Belarusian healthcare institutions, (ii) were research staff with no direct involvement in clinical care, or (iii) were students pursuing undergraduate healthcare education.

This study employed a snowball sampling method for data collection from the target population. Invitations to participate in the study were disseminated through various social media groups of Belarusian medical doctors and allied HCPs, especially in Minsk, e.g., Facebook, VK, Instagram, and Viber, as well as some medical centres. Participants were asked to recommend completing the questionnaire to their colleagues and acquaintances.

The required minimum sample size for this investigation was calculated using Epi Info^TM^ version 7.2.5 (CDC, Atlanta, GA, USA, 2021) [18]. The computation took into account the following parameters: (i) a confidence level (CI) of 95%; (ii) a permissible margin of error set at 5%; (iii) an estimated target population exceeding 120,000; and (iv) an anticipated incidence rate of the principal outcome—namely, the acceptance of the mpox vaccine—of 10% [19,20].

To infer a relationship between hypothesised demographic and anamnestic predictors and the prevailing intentions of Belarusian HCPs to accept the mpox vaccine, no fewer than 138 valid responses were required. Overall, 152 HCWs clicked on the survey link or partially completed the survey, of which only 8 were excluded because they did not meet the inclusion criteria or their answers were considered as careless or invalid.

### 2.3. Instrument

The study used an SAQ comprised of 55 structured questions classified into eight categories: (i) sociodemographic attributes (gender, sexual orientation, marital status, age, the presence of children under 18, profession, region, etc), (ii) medical history (presence of chronical diseases, usage of medical treatment on a regular basis, vaccination against COVID-19, influenza, and smallpox), (iii) mpox information sources (information on mpox during studies, information source used for information on monkeypox, trust in the different information sources), (iv) perceived knowledge of mpox (knowledge of epidemiology, clinical symptoms, ways of transmission and risk factors, vaccination, and treatment), (v) factual knowledge of mpox (incubation period, fatal levels, endemic regions, symptoms, diagnostic criteria, localisation of clinical symptoms, ways of transmission, vaccination, and treatment), (vi) perceptions of the mpox vaccine based on the HBM (including perceived susceptibility, perceived severity, perceived benefits, perceived barriers, and cues to action), (vii) intent to accept and recommend the mpox vaccine, and (viii) willingness to pay for the mpox vaccine (amount of money which is optimal and respondents ready to pay). The draft SAQ was informed by earlier studies on mpox among HCPs and other demographics [21,22,23,24]. Experts in public health, infectious diseases, and psychology evaluated and provided feedback on a draft questionnaire to ensure its validity. Confirmatory factor analysis suggested that the final questionnaire had good construct validity, and full psychometric properties were described in detail earlier [19]. The present SAQ was translated into the Russian language by two independent translators who had experience with medical terminology (forward translation); later, the two Russian versions were compared and evaluated by a committee of three native Russian speakers (expert panel review) to produce a final Russian version.

### 2.4. Measures

Fifteen multiple-choice questions (MCQs) were employed to evaluate the factual knowledge of the participants regarding mpox. Each knowledge domain was represented by three items. Ten MCQs had a unique correct answer, resulting in a binary scoring system (true = 1; false = 0). For the remaining five MCQs, which had multiple correct answers, a three-tiered rating was adopted (advanced knowledge = 2; acceptable knowledge = 1; no knowledge = 0).

Additionally, participants’ confidence in mpox information sources was gauged on a 7-point Likert scale, ranging from ‘extremely unreliable’ (1) to ‘extremely reliable’ (7). In the same manner, a 5-point Likert scale was used to rate the perceived knowledge of mpox, HBM components, and attitudes towards the mpox vaccine, ranging from ‘strongly disagree’ (1) to ‘strongly agree’ (5).

The item for assessing acceptance of the mpox vaccine was expressed as: “I am willing (interested) to receive a vaccine against human monkeypox in the near future”. This was evaluated on a 5-point Likert scale, which ranged from ‘strongly disagree’ (1) to ‘strongly agree’ (5). Acceptance of the mpox vaccine was inferred if a participant selected either “agree” or “strongly agree”. Conversely, vaccine hesitancy was assumed if the participant chose “not sure”, while rejection of the mpox vaccine was ascertained if the participant opted for “disagree” or “strongly disagree”.

### 2.5. Ethics

The study protocol underwent review and received approval from the Ethical Committee of the Faculty of Medicine at Masaryk University on 19 September 2022, under reference number 73/2022. Adherence to the Declaration of Helsinki and the General Data Protection Regulation (GDPR) of the European Union (EU) was maintained during all data gathering and processing activities [25,26]. The first page of the digital SAQ presented a brief explanation of the study objectives, inclusion and exclusion criteria, participation benefits and risks, and the consent statement. Participants had to provide their consent digitally at the beginning of the survey as a prerequisite to participation in the study. 

### 2.6. Analyses

The execution of all statistical analyses was facilitated through SPSS version 28.0 (SPSS Inc., Chicago, IL, USA, 2020) [27]. Firstly, the Shapiro–Wilk test, at a significance level (*p*) ≤ 0.05, was utilised to verify the normal distribution of the numerical and ordinal variables. Subsequently, categorical variables, including aspects such as gender, sexual orientation, and profession, were described using frequencies (*n*) and percentages (%). Likewise, ordinal and numerical variables, such as confidence levels, perceptions, and knowledge scores, were summarised using means and standard deviations (*µ* ± *SD*). Inferential statistical analyses were conducted using the chi-squared (*χ^2^*), Fisher’s exact, Analysis of Variance (ANOVA), Kruskal–Wallis (*H*), and Mann–Whitney (*U*) tests, all with a significance threshold of *p* ≤ 0.05.

## 3. Results

### 3.1. Demographic and Anamnestic Characteristics

Out of the 144 participating HCPs, 136 (94.4%) were females, and 133 (92.4%) were heterosexuals. The participants’ mean age was 31.89 ± 7.17 years old, 111 (77.1%) were married, and 87 (60.4%) had children. Most participants were medical HCPs (77.8%), and only 22.2% would be potentially involved in clinical care provision to mpox cases (Table 1).

Overall, 60 (41.7%) participants reported suffering from at least one non-communicable disease. Bowel disease was the most commonly reported (14.6%), followed by thyroid disease (11.8%), ophthalmologic conditions (9%), allergies (7.6%), cardiovascular disease (5.6%), renal disorders (4.9%), and chronic hypertension (3.5%). Furthermore, 33 (22.9%) participants reported receiving at least one medication regularly. Thyroid hormones (7.6%) were the most commonly reported medications, followed by other hormonal drugs (5.6%), antihypertensives (3.5%), contraceptives (3.5%), antidiabetics (2.1%), and antihistamines (1.4%).

The majority of participants (80.6%) reported receiving at least one dose of COVID-19 vaccines; 4 (2.8%) received five doses or more, 22 (15.3%) received four doses, 42 (29.2%) received three doses, 44 (30.6%) received two doses, and 4 (2.8%) received one dose only. Most participants also reported receiving the influenza vaccine (81.3%), out of which 58.1% were vaccinated during the last three years. There was a significant association between the influenza vaccination and mpox vaccine hesitancy (*p*. = 0.004). On the other hand, the majority of participants (81.3%) did not receive any smallpox vaccines. Additionally, smallpox vaccination is not routinely provided to HCPs in Belarus now (Table 2).

### 3.2. Mpox Information Sources

Only one-fifth of the respondents (20.8%) reported learning about mpox through their undergraduate curricula. The most commonly utilised information source for learning about the 2022 mpox outbreak was social media platforms, e.g., Facebook, VK, and Instagram (62.5%), followed by the WHO (58.3%), the Ministry of Health (MoH) (47.2%), news portals (33.3%), professional societies (25.7%), and public health authorities (22.2%). The average number of utilised information sources was 3.1 ± 1.8 (0–10).

When asked about their confidence in utilised information sources, the US CDC was the most trusted, with a mean score of 6.1 ± 0.4 (1–7), followed by professional societies (6.0 ± 0.6), the ECDC (5.9 ± 0.5), scholarly journals (5.9 ± 0.5), and the WHO (5.8 ± 0.7). Notably, the least trusted sources were the MoH (4.5 ± 1.4), the pharmaceutical industry (4.6 ± 1.0), and social media (4.7 ± 1.0) (Table 3).

### 3.3. Mpox-Related Perceived Knowledge

When asked about their perceived knowledge on a 5-point Likert scale ranging from (1 = strongly disagree) to (5 = strongly agree), the epidemiology domain received a mean score of 2.6 ± 0.9, clinical presentation (2.8 ± 1.0), transmission pathways (3.0 ± 1.0), preventive techniques (2.5 ± 0.9), and treatment modalities (2.4 ± 0.9). There was no statistically significant difference in mpox vaccine attitudes across the perceived knowledge scores (Table 4).

On analysing the predictors of mpox-related perceived knowledge, the overall mean score was 13.3 ± 4.0, and it ranged between 5 and 25. The history of influenza vaccination was significantly associated with mpox-related factual knowledge (*p*. = 0.010), and those who received the influenza vaccine had a mean score of 13.7 ± 3.7, while those who were not vaccinated had a mean score of 11.2 ± 4.8. Notably, the utilisation of public health institutions, the ECDC, US CDC, and WHO, professional associations, scientific journals, and news portals as information sources were significantly associated (*p*. = 0.013, 0.002, 0.002, <0.001, =0.043, 0.006 and 0.039, respectively) with the highest overall scores of perceived knowledge (14.9 ± 3.2), (15.6 ± 2.5), (15.6 ± 3.0), (14.5 ± 3.0), (14.5 ± 3.4), (14.8 ± 3.9) and (14.3 ± 3.3) (Table S1).

### 3.4. Mpox-Related Factual Knowledge

About 59% and 59.7% of the participants correctly answered the questions regarding the mpox incubation period and its case–fatality ratio, respectively. The most correctly answered question about epidemiology was the endemic region question, as 70.8% of the participants knew that mpox used to be endemic in sub-Saharan Africa.

As high as 91% of the participants were aware of at least one clinical symptom of mpox, while 52.8% were able to tell the differential diagnosis between mpox and smallpox. Also, 71.5% knew at least one of the mpox lesion locations. Vertical transmission was the only item which was significantly (*p*. = 0.036) associated with mpox vaccine attitudes.

The vast majority of participants (92.4%) knew at least one of the mpox transmission pathways, while only 36.1% and 66% correctly answered the questions regarding vertical transmission and sexual transmission, respectively.

Only 23.6% were aware that there was an available vaccine against mpox, 74.3% knew at least one of the recommended groups to receive pre-exposure prophylaxis (PrEP), and 70.8% knew that the smallpox vaccine is the only vaccine that provides cross-immunisation against mpox.

Only 19.4% were aware that there was an available treatment for mpox, 17.4% knew at least one of the mpox medications, and 38.2% knew that mpox caused a mild and self-limiting clinical course (Table S2).

On analysing the predictors of mpox-related factual knowledge, the overall mean score was 10.5 ± 3.9, and it ranged between 0 and 20. The history of influenza vaccination was significantly associated with mpox-related factual knowledge (*p*. = 0.004), and those who received the influenza vaccine had a mean score of 11.0 ± 3.6, while those who were not vaccinated had a mean score of 8.0 ± 4.7. Notably, the utilisation of the ECDC, US CDC, and WHO as information sources were significantly associated (*p*. = 0.028, <0.001, and <0.001, respectively) with the highest overall scores of factual knowledge (12.1 ± 3.8), (13.2 ± 3.0), and (11.6 ± 3.7) (Table S3).

### 3.5. Correlation of Perceived and Factual Knowledge

On performing correlation analysis between perceived and factual knowledge domains, epidemiology had a moderate correlation (*Spearman’s rho* = 0.428). Similarly, clinical presentation, transmission pathways, and preventive techniques had a moderate correlation (*Spearman’s rho* = 0.344, 0.333, and 0.393), respectively. However, treatment modalities had a poor correlation (*Spearman’s rho* = 0.282) (Table 5).

### 3.6. Mpox Vaccine Acceptance

Overall, more than half of the participants (51.4%) indicated their acceptance to receiving vaccination against mpox; 30.6% and 18.1% were unsure and rejected mpox vaccination, respectively. Among the five domains of the health belief model (HBM), perceived benefits (10.6 ± 2.6) and cues to action (10.2 ± 2.4) had the highest mean scores (range from 3 to 15).

The item “I believe that I am susceptible to get infected by monkeypox due to my occupation” was significantly associated with mpox vaccine attitudes (*p*. = 0.016), as the rejection, hesitancy, and acceptance groups had mean scores of (2.4 ± 1.2), (2.8 ± 1.0), and (3.1 ± 1.1), respectively. Similarly, the items “when I get vaccinated against human monkeypox, I will be protected from getting infected”, “when I get vaccinated against human monkeypox, I will be protected from serious complications of the disease”, and “when I get vaccinated against human monkeypox, I will protect my patients, family, and friends from infection” were significantly associated with mpox vaccine attitudes (*p*. < 0.001, <0.001, and <0.001, respectively).

The item “I feel concerned about availability (access) of monkeypox vaccines” was significantly associated with mpox vaccine attitudes (*p*. < 0.001), as the rejection, hesitancy, and acceptance groups had mean scores of (2.6 ± 1.1), (3.1 ± 1.0), and (3.8 ± 1.1), respectively. Similarly, the items “the chances of me getting vaccinated against monkeypox will increase if public health authorities recommend it” and “the chances of me getting vaccinated against monkeypox will increase if reliable evidence about their effectiveness/safety is available” were significantly associated with mpox vaccine attitudes (*p*. < 0.001, and <0.001, respectively) (Table 6).

### 3.7. Correlates of Mpox Vaccine Acceptance

**Figure 1:**
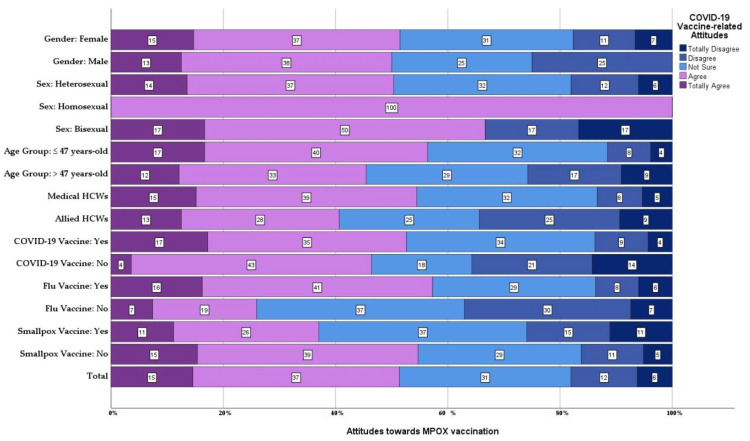
Attitudes of Belarusian HCPs towards mpox vaccination, stratified by gender, sex, age, profession and other vaccine history, October 2022, (*n* = 144).

On evaluating mpox vaccine attitudes with a 5-point Likert scale, ranging from (1 = strongly disagree) to (5 = strongly agree), the mean score was 3.4 ± 1.1 (1–5). There was no statistically significant difference across gender, sexual orientation, age group, having children, profession, the status of providing care, chronic illnesses, medications, COVID-19 vaccination status, number of doses, smallpox vaccination, or utilised information sources (Figure 1).

On the other hand, those who had ever received the influenza vaccine were more in favour of the mpox vaccine (3.5 ± 1.0) than those who had never been vaccinated against influenza (Table S4).

The non-parametric correlation between HBM domains and mpox vaccine acceptance indicated perceived benefits (*Spearman’s rho* = 0.451) and cues to action (*Spearman’s rho* = 0.349) had fair correlations. Other domains, i.e., perceived susceptibility, perceived severity, and perceived barriers, were negligible in terms of correlation with mpox vaccine attitudes (Table 7).

### 3.8. Mpox Vaccine Recommendations and Willingness to Pay

When surveying how much respondents would consent to pay for the mpox vaccine shot from their own pockets, we found that 21.5% hoped to receive it for free. Meanwhile, 28.5% were amenable to a price tag of less than BYN 24.7 per dose, and 47.2% were fine with a price ranging between BYN 24.71 to 121.10 per dose.

Furthermore, when queried about the ideal public pricing for the mpox vaccine, we found that 35.4% hoped it would be offered for free. Conversely, 39.6% suggested a price less than BYN 24.7 per dose would be appropriate, while 25% were comfortable with a price range of BYN 24.71 to 121.10 per dose (Table 8).

## 4. Discussion

This cross-sectional study aimed to understand knowledge and attitudes towards mpox and its vaccine among Belarusian HCPs. The study found that a majority of the participants were aware of mpox epidemiology and its clinical symptoms, but only a minority were aware of the available vaccine and treatment options. Interestingly, the study revealed a significant association between influenza vaccination history and mpox-related knowledge. Additionally, the study found that more than half of the participants (51.4%) were willing to receive the mpox vaccine, with perceived benefits and cues to action playing a significant role in their decision. On the contrary, in China 90.12% expressed their willingness for vaccination (vaccine hesitancy rate = 9.88%) [28]. However, there was a notable level of hesitancy (30.6%) and rejection (18.1%) towards the mpox vaccine, indicating the need for targeted interventions to address these concerns. Similar levels of hesitancy, 33.3%, and resistance, 37.8%, were found among HCPs in Jordan [29].

Social media platforms were the most commonly utilised information source to learn about mpox in our sample (62.5%). Likewise, Berdida, 2023, found that social media, particularly Facebook, was the most common mpox information source among the general Filipino population (78.61%), followed by mass media (15.13%) and other people (6.26%) [30]. Additionally, Alsharani et al., 2022, found that social media was the most common source used by the general Saudi population (75%), followed by TV and radio (45.6%), family or friends (15.6%), and healthcare provider (13.8%) [31]. Scholarly journals and professional medical societies were reportedly utilised by 20.8% and 25.7% of the participants as information sources. On the other hand, only 5.6% and 12% of Czech HCPs utilised scholarly journals and professional medical societies to learn about mpox [19]. 

On a 7-point Likert scale, our participants exhibited the lowest confidence levels towards the Ministry of Health (4.5 ± 1.4) and the pharmaceutical industry (4.6 ± 1.0). This finding is in line with the global trends observed during infectious disease outbreaks. For instance, a study by Baekkeskov, 2016, on the 2009 H1N1 influenza pandemic highlighted the role of public health specialists, elected leaders, and organisations in shaping national responses, which can be influenced by the level of trust in these entities [32]. Similarly, Ozisik et al., 2016, discussed the importance of HCPs in vaccination and the barriers to vaccination, including mistrust in health authorities and the pharmaceutical industry [33]. Paul and Loer, 2019, also emphasised the need for a nuanced discourse on vaccination policy, acknowledging the role of trust in health authorities [34]. These findings underscore the importance of building and maintaining trust in health authorities and the pharmaceutical industry, especially during infectious disease outbreaks and pandemics.

Among our participants, international health organisations such as the US CDC (6.1 ± 0.4), the ECDC (5.9 ± 0.5), and the WHO (5.8 ± 0.7) were held in high regard, overshadowing local sources. This preference could be a reflection of the unfavourable political climate in Belarus [35]. In such circumstances, HCPs often turn to international bodies for reliable information. This trend is not confined to Belarus; research indicates that local health authorities are often viewed with scepticism in countries with similar political environments, while HCPs favour international health organisations. The systematic review of Larson et al., 2018, emphasised that vaccine acceptance heavily depends on public trust and confidence in the safety and efficacy of vaccines, the health system, HCPs, and the wider vaccine research community [36]. However, it is worth noting that this reliance on international sources could potentially lead to a lack of context-specific information that caters to the unique needs and circumstances of the Belarusian population [37]. In the USA, the general public found healthcare professionals and officials, but also known doctors and researchers with a large online following, among the most trusted sources regarding the monkeypox outbreak, which once again highlights the importance of knowledge among HCPs being trusted by general population [38].

While the correlation between perceived and factual knowledge had the highest score in the domain of epidemiology (*Spearman’s rho* = 0.428), it had the lowest score in the domain of treatments (*Spearman’s rho* = 0.282). Similarly, Riad et al., 2022, found that the lowest correlation coefficient among Czech HCPs was found in the domain of treatments (*Spearman’s rho* = 0.197) [19]. One of the explanations for this weak correlation in the domain of treatments is the increased perceived knowledge (2.4 ± 0.9 [1–5]) compared with factual knowledge (0.8 ± 0.9 [0–4]). The same was found in Czechia; the perceived knowledge mean (2.8 ± 1.0 [1–5]) was higher than the factual knowledge mean (0.9 ± 0.9 [0–4]) of mpox treatments [19].

The discrepancy between perceived and factual knowledge in the domain of treatments, as highlighted in our study, is a reflection of the knowledge fallacy, which happens when perceived knowledge is overestimated compared to factual knowledge [39,40,41,42,43,44]. This fallacy can be particularly problematic in healthcare settings, where accurate knowledge and its application are critical for effective patient care. Sherbino et al., 2012, highlighted that those cognitive biases resulting from knowledge illusion might lead to diagnostic errors [45].

Most of our participants (70.8%) were aware that sub-Saharan Africa used to be the endemic region for mpox before the 2022 outbreak. Similarly, 66% and 58.3% of Kuwaiti nurses and physicians were aware of this fact [46]. In Lebanon and Jordan, 77.4% and 64.5% of HCPs were also aware that mpox was prevalent in western and central Africa [47,48]. Contrarily, only 24.8% of Saudi physicians knew this fact [49].

In comparison with Czech HCPs, our participants had more correct answers to the items of differential diagnosis (40.5% vs. 52.8%), vertical transmission (23.5% vs. 36.1%), pre-exposure prophylaxis (59.5% vs. 74.3%), and cross-immunisation (38.7% vs. 70.8%), respectively [19]. On the other hand, Czech HCPs had more correct answers to the items of vaccine availability (33.7% vs. 23.6%) and prognosis (48.4% vs. 38.2%), respectively [19].

In our study, we found that the use of the ECDC, US CDC, and WHO as information sources was significantly associated (*p*. = 0.028, <0.001, and <0.001, respectively) with the highest overall scores of monkeypox factual knowledge (12.1 ± 3.8), (13.2 ± 3.0), and (11.6 ± 3.7). Similarly, Riad et al., 2022, indicated that among Czech HCPs, the US CDC had the highest factual knowledge score (15.2 ± 2.3), followed by the ECDC (12.4 ± 4.6) and the WHO (12.3 ± 3.2) [19].

More than half of the participants (51.4%) indicated their acceptance of receiving the vaccination against mpox, while 30.6% and 18.1% were unsure and rejected it, respectively. Likewise, Ricco et al., 2022, found that 64.4% of Italian physicians were in favour of receiving the variola vaccine [21]. Also, Sahin et al., 2022, reported that 31.4%, 38.5%, and 30% of Turkish physicians were willing, hesitant, and not willing to receive vaccination against mpox, respectively [50]. In Algeria, 38.7% of HCPs were in favour of mpox vaccination if recommended for free [51]. In contrast, only 8.8% of Czech HCPs were open to receiving vaccination against mpox, and 46.3% and 44.9% were unsure and resistant to mpox vaccination [52].

On exploring the psychological predictors of mpox vaccination acceptance, perceived benefits (*Spearman’s rho* = 0.451) and cues to action (*Spearman’s rho* = 0.349) had moderate correlations with mpox vaccine acceptance. These findings are in agreement with what was found among Czech HCPs, where the cues to action (*Spearman’s rho* = 0.569) and perceived benefits (*Spearman’s rho* = 0.372) had moderate correlations with mpox vaccine acceptance [19]. In Turkey, physicians who were more worried about mpox compared with COVID-19 were more likely to accept mpox vaccination [50]. Also, Turkish physicians with higher factual mpox knowledge scores had higher odds of accepting mpox vaccination [50]. Among a Peruvian sample, fear of mpox played an important role, both directly and indirectly, in the intention to be vaccinated against mpox, having conspiratorial beliefs about mpox as a mediating variable [53].

Regarding the demographic and anamnestic correlates of mpox vaccine acceptance, there was no statistically significant difference across gender, sexual orientation, age group, having children, profession, the status of providing care, chronic illnesses, medications, COVID-19 vaccination status, number of doses, smallpox vaccination, or utilised information sources. A recent systematic review by Ulloque-Badaracco et al., 2022, revealed that the global prevalence of mpox vaccination acceptance was 56.0%, with significant variations across different populations and regions [54]. The study highlighted that the LGBTI community had the highest acceptance rate at 84%, followed by healthcare workers at 63%, and the general population at 43%, underscoring the necessity for public health strategies to be tailored to increase vaccination rates in these specific groups [54]. In Ghana, Ghazy et al., 2023, found that factors such as gender, residential location, prior refusal of the COVID-19 vaccine, trust in vaccination, and a sense of communal responsibility significantly influenced the decision to accept the mpox vaccine [55]. COVID-19 vaccine eagerness and having concerns about mpox epidemics were associated with favourable attitudes toward mpox vaccination in HCPs in France [56].

The only important medical history factor was the history of seasonal influenza vaccination, as those who had ever received the influenza vaccine were more in favour of the mpox vaccine (3.5 ± 1.0) than those who had never been vaccinated against influenza. Likewise, influenza vaccination history was significantly associated with mpox vaccine acceptance (13% vs. 6.2%; *Sig* = 0.031) among Czech HCPs [19].

### 4.1. Study Strengths

This research represents the inaugural study investigating the understanding of mpox and attitudes towards vaccination among HCPs in Belarus. It is important to highlight the current scarcity of research focusing on attitudes towards vaccination and awareness of infectious diseases in Eastern Europe, and Belarus in particular. The HBM serves as a robust framework in this study, enabling the design of bespoke interventions. These interventions, whether they are educational or communication-focused, aim to enhance the willingness for vaccination among Belarusian HCPs.

### 4.2. Study Limitations

This study is not without its limitations. Firstly, there was a gender imbalance in the sample, with a significant majority of respondents being women (94.4%). While this may seem skewed, it is reflective of the gender distribution within the Belarusian healthcare system, where 85% of HCPs are women [57]. Secondly, the snowball sampling technique employed in this study may have introduced self-selection and reporting biases. This is because those with an interest in mpox, infectious diseases, or public health emergencies may have been more likely to participate in the study. Thirdly, the study may have included healthcare workers who perceive their risk of acquiring monkeypox as low or negligible due to their specific roles and exposure opportunities. These factors should be considered when interpreting the results. Lastly, while the sample size was calculated based on the primary outcome of mpox vaccine acceptance, it is important to note that other aspects of the study, such as mpox knowledge and vaccine perception, might require a larger sample size for more representative and valid findings. This should be considered when interpreting the results related to these secondary outcomes. These factors should be taken into consideration when interpreting the results.

### 4.3. Study Implications

The findings of this study underscore the necessity for targeted interventions to enhance mpox knowledge and vaccination readiness among Belarusian HCPs. Given the results of the HBM, it is recommended that health policymakers and practitioners in Belarus develop tailored educational and communication strategies to address this need. 

Furthermore, considering the high proportion of women in the Belarusian healthcare system, future interventions should also consider gender-specific approaches to effectively address knowledge gaps and attitudes towards vaccination. The gender imbalance and potential self-selection bias in the study sample also highlight the importance of inclusive and diverse recruitment strategies in future research. Lastly, the study’s findings should catalyse further research into vaccination attitudes and infectious disease awareness in Eastern Europe, particularly in Belarus.

## 5. Conclusions

This study examined mpox knowledge and vaccination attitudes among Belarusian healthcare professionals. While most were informed about mpox epidemiology and symptoms, few were aware of available vaccines and treatments. Over half were open to the mpox vaccine (51.4%), yet significant hesitancy and rejection were noted. Social media was the primary mpox information source, with low trust in local health authorities. The study revealed a gap between perceived and actual knowledge, particularly regarding treatment options. These findings underscore the need for targeted interventions to strengthen mpox knowledge and vaccine acceptance.

## Figures and Tables

**Table 1:** Demographic Characteristics of Belarusian Healthcare Professionals Participating in the mpox Survey, October 2022, (*n* = 144).

Variable	Outcome	Mpox VAX Rejection (*n* = 26)	Mpox VAX Hesitancy (*n* = 44)	Mpox VAX Acceptance (*n* = 74)	Total (*n* = 144)	*p.*
Gender	Female	24 (92.3%)	42 (95.5%)	70 (94.6%)	136 (94.4%)	0.789
	Male	2 (7.7%)	2 (4.5%)	4 (5.4%)	8 (5.6%)	
Sex	Heterosexual	24 (92.3%)	42 (95.5%)	67 (90.5%)	133 (92.4%)	0.482
	Homosexual	0 (0%)	0 (0%)	1 (1.4%)	1 (0.7%)	
	Bisexual	2 (7.7%)	0 (0%)	4 (5.4%)	6 (4.2%)	
	Other	0 (0%)	2 (4.5%)	2 (2.7%)	4 (2.8%)	
Marital Status	Single	5 (19.2%)	7 (15.9%)	14 (18.9%)	26 (18.1%)	0.008
	Married	16 (61.5%)	36 (81.8%)	59 (79.7%)	111 (77.1%)	
	Widowed	0 (0%)	0 (0%)	1 (1.4%)	1 (0.7%)	
	Divorced	5 (19.2%)	1 (2.3%)	0 (0%)	6 (4.2%)	
Age Group	≤31 years old	9 (34.6%)	25 (56.8%)	44 (59.5%)	78 (54.2%)	0.084
	>31 years old	17 (65.4%)	19 (43.2%)	30 (40.5%)	66 (45.8%)	
Have Children?	No	8 (30.8%)	16 (36.4%)	33 (44.6%)	57 (39.6%)	0.404
	Yes	18 (69.2%)	28 (63.6%)	41 (55.4%)	87 (60.4%)	
Profession	Medical HCPs	15 (57.7%)	36 (81.8%)	61 (82.4%)	112 (77.8%)	0.025
	Allied HCPs	11 (42.3%)	8 (18.2%)	13 (17.6%)	32 (22.2%)	
Provide Care?	No	21 (80.8%)	37 (84.1%)	54 (73%)	112 (77.8%)	0.343
	Yes	5 (19.2%)	7 (15.9%)	20 (27%)	32 (22.2%)	

The Chi-squared test (*χ^2^*) and Fisher’s exact test were used with a significance level (*p*.) ≤ 0.05.

**Table 2:** Medical History of Belarusian Healthcare Professionals Participating in the mpox Survey, October 2022, (*n* = 144).

Variable	Outcome	Mpox VAX Rejection (*n* = 26)	Mpox VAX Hesitancy (*n* = 44)	Mpox VAX Acceptance (*n* = 74)	Total (*n* = 144)	*p.*
Chronic Diseases	Bowel Disease	5 (19.2%)	6 (13.6%)	10 (13.5%)	21 (14.6%)	0.773
	Thyroid Disease	3 (11.5%)	5 (11.4%)	9 (12.2%)	17 (11.8%)	1.000
	Ophthalmologic Disease	1 (3.8%)	4 (9.1%)	8 (10.8%)	13 (9%)	0.680
	Allergy	2 (7.7%)	2 (4.5%)	7 (9.5%)	11 (7.6%)	0.643
	Cardiovascular Disease	1 (3.8%)	2 (4.5%)	5 (6.8%)	8 (5.6%)	1.000
	Renal Disorders	2 (7.7%)	1 (2.3%)	4 (5.4%)	7 (4.9%)	0.595
	Chronic Hypertension	2 (7.7%)	0 (0%)	3 (4.1%)	5 (3.5%)	0.158
	Neurologic Disease	2 (7.7%)	1 (2.3%)	0 (0%)	3 (2.1%)	0.062
	Blood Disease	0 (0%)	1 (2.3%)	1 (1.4%)	2 (1.4%)	1.000
	Cancer	0 (0%)	0 (0%)	2 (2.7%)	2 (1.4%)	0.684
	Rheumatoid Arthritis	1 (3.8%)	1 (2.3%)	0 (0%)	2 (1.4%)	0.235
	Asthma	0 (0%)	0 (0%)	1 (1.4%)	1 (0.7%)	1.000
	Diabetes Mellitus type-I	0 (0%)	0 (0%)	1 (1.4%)	1 (0.7%)	1.000
	Diabetes Mellitus type-II	1 (3.8%)	0 (0%)	0 (0%)	1 (0.7%)	0.181
	Hepatologic Disease	1 (3.8%)	0 (0%)	0 (0%)	1 (0.7%)	0.181
	Total	12 (46.2%)	19 (43.2%)	29 (39.2%)	60 (41.7%)	0.801
Medications	Thyroid Hormones	2 (7.7%)	6 (13.6%)	3 (4.1%)	11 (7.6%)	0.155
	Hormonal Drugs	0 (0%)	4 (9.1%)	4 (5.4%)	8 (5.6%)	0.297
	Antihypertensives	1 (3.8%)	1 (2.3%)	3 (4.1%)	5 (3.5%)	1.000
	Contraceptives	1 (3.8%)	1 (2.3%)	3 (4.1%)	5 (3.5%)	1.000
	Antidiabetics	1 (3.8%)	1 (2.3%)	1 (1.4%)	3 (2.1%)	0.756
	Antihistamines	0 (0%)	1 (2.3%)	1 (1.4%)	2 (1.4%)	1.000
	Immunosuppressives	1 (3.8%)	1 (2.3%)	0 (0%)	2 (1.4%)	0.235
	Anticoagulants	1 (3.8%)	0 (0%)	0 (0%)	1 (0.7%)	0.181
	NSAIDs	0 (0%)	0 (0%)	1 (1.4%)	1 (0.7%)	1.000
	Other Medications	0 (0%)	2 (15.4%)	2 (12.5%)	4 (12.1%)	1.000
	Total	4 (15.4%)	13 (29.5%)	16 (21.6%)	33 (22.9%)	0.368
COVID-19 Vaccine	One Dose	1 (3.8%)	1 (2.3%)	2 (2.7%)	4 (2.8%)	1.000
	Two Doses	5 (19.2%)	17 (38.6%)	22 (29.7%)	44 (30.6%)	0.229
	Three Doses	7 (26.9%)	12 (27.3%)	23 (31.1%)	42 (29.2%)	0.873
	Four Doses	2 (7.7%)	9 (20.5%)	11 (14.9%)	22 (15.3%)	0.369
	≥Five Doses	1 (3.8%)	0 (0%)	3 (4.1%)	4 (2.8%)	0.399
	Total	16 (61.5%)	39 (88.6%)	61 (82.4%)	116 (80.6%)	0.018
Flu Vaccine	No	10 (38.5%)	10 (22.7%)	7 (9.5%)	27 (18.8%)	0.004
	Yes	16 (61.5%)	34 (77.3%)	67 (90.5%)	117 (81.3%)	
Recent Flu Vaccine	No	6 (37.5%)	13 (38.2%)	30 (44.8%)	49 (41.9%)	0.762
	Yes	10 (62.5%)	21 (61.8%)	37 (55.2%)	68 (58.1%)	
Smallpox Vaccine	No	19 (73.1%)	34 (77.3%)	64 (86.5%)	117 (81.3%)	0.199
	Yes	7 (26.9%)	10 (22.7%)	10 (13.5%)	27 (18.8%)	

The Chi-squared test (*χ^2^*) and Fisher’s exact test were used with a significance level (*p*.) ≤ 0.05.

**Table 3:** Information Sources of Belarusian Healthcare Professionals Participating in the mpox Survey, October 2022, (*n* = 144).

Variable	Outcome	Mpox VAX Rejection (*n* = 26)	Mpox VAX Hesitancy (*n* = 44)	Mpox VAX Acceptance (*n* = 74)	Total (*n* = 144)	*p.*
Undergraduate Curriculum	No	19 (73.1%)	36 (81.8%)	59 (79.7%)	114 (79.2%)	0.675
	Yes	7 (26.9%)	8 (18.2%)	15 (20.3%)	30 (20.8%)	
Commonly Utilised Information Sources	Social Media	12 (46.2%)	25 (56.8%)	53 (71.6%)	90 (62.5%)	0.045
	WHO	15 (57.7%)	24 (54.5%)	45 (60.8%)	84 (58.3%)	0.798
	Ministry of Health	9 (34.6%)	21 (47.7%)	38 (51.4%)	68 (47.2%)	0.338
	News Portals	7 (26.9%)	13 (29.5%)	28 (37.8%)	48 (33.3%)	0.487
	Professional Societies	10 (38.5%)	9 (20.5%)	18 (24.3%)	37 (25.7%)	0.232
	Public Health Authorities	8 (30.8%)	10 (22.7%)	14 (18.9%)	32 (22.2%)	0.456
	Scholarly Journals	4 (15.4%)	8 (18.2%)	18 (24.3%)	30 (20.8%)	0.548
	ECDC	4 (15.4%)	7 (15.9%)	13 (17.6%)	24 (16.7%)	1.000
	US CDC	3 (11.5%)	5 (11.4%)	14 (18.9%)	22 (15.3%)	0.528
	Pharmaceutical Industry	1 (3.8%)	4 (9.1%)	5 (6.8%)	10 (6.9%)	0.752
	Total (0–10)	2.8 ± 1.6	2.9 ± 1.7	3.3 ± 1.9	3.1 ± 1.8	0.337
Level of Confidence (1–7)	US CDC	6.3 ± 0.6	6.0 ± 0.0	6.1 ± 0.4	6.1 ± 0.4	0.427
	Professional Societies	6.1 ± 0.6	6.0 ± 0.5	6.0 ± 0.6	6.0 ± 0.6	0. 887
	ECDC	6.0 ± 0.8	6.0 ± 0.0	5.9 ± 0.6	5.9 ± 0.5	0.744
	Scholarly Journals	5.5 ± 0.6	5.9 ± 0.4	6.1 ± 0.4	5.9 ± 0.5	0.072
	WHO	5.8 ± 0.7	6.0 ± 0.6	5.8 ± 0.8	5.8 ± 0.7	0.478
	Public Health Authorities	4.5 ± 1.4	5.1 ± 0.9	4.8 ± 1.3	4.8 ± 1.2	0.700
	News Portals	4.4 ± 1.5	4.9 ± 0.9	4.6 ± 0.8	4.7 ± 0.9	0.747
	Social Media	4.9 ± 1.2	4.7 ± 0.9	4.6 ± 1.0	4.7 ± 1.0	0.425
	Pharmaceutical Industry	4.0 ± 0.0	4.5 ± 0.6	4.8 ± 1.3	4.6 ± 1.0	0.696
	Ministry of Health	4.1 ± 1.8	5.0 ± 0.9	4.3 ± 1.4	4.5 ± 1.4	0.205

The Chi-squared test (*χ^2^*), Fisher’s exact test and Kruskal–Wallis (*H*) were used with a significance level (*p*.) ≤ 0.05.

**Table 4:** Knowledge of Belarusian Healthcare Professionals Participating in the mpox Survey, October 2022, (*n* = 144).

Variable	Outcome	Mpox VAX Rejection (*n* = 26)	Mpox VAX Hesitancy (*n* = 44)	Mpox VAX Acceptance (*n* = 74)	Total (*n* = 144)	*p.*
Perceived Knowledge	Epidemiology: (1–5)	2.7 ± 0.8	2.4 ± 0.9	2.6 ± 0.9	2.6 ± 0.9	0.317
	Clinical Presentation: (1–5)	3.0 ± 0.8	2.6 ± 1.1	2.9 ± 0.9	2.8 ± 1.0	0.399
	Transmission: (1–5)	3.2 ± 1.0	2.7 ± 1.1	3.1 ± 1.0	3.0 ± 1.0	0.087
	Prevention: (1–5)	2.5 ± 0.9	2.4 ± 0.9	2.6 ± 0.9	2.5 ± 0.9	0.546
	Treatment: (1–5)	2.3 ± 0.8	2.3 ± 1.0	2.5 ± 0.9	2.4 ± 0.9	0.444
Factual Knowledge: Epidemiology	Incubation Period	13 (50%)	26 (59.1%)	46 (62.2%)	85 (59%)	0.555
	Case-Fatality Ratio	13 (50%)	23 (52.3%)	50 (67.6%)	86 (59.7%)	0.140
	Endemic Region	20 (76.9%)	30 (68.2%)	52 (70.3%)	102 (70.8%)	0.731
	Total (0–3)	1.8 ± 1.0	1.8 ± 1.0	2.0 ± 1.0	1.9 ± 1.0	0.408
Factual Knowledge: Clinical Presentation	Clinical Symptoms	24 (92.3%)	41 (93.2%)	66 (89.2%)	131 (91%)	0.800
	Differential Diagnosis	14 (53.8%)	25 (56.8%)	37 (50%)	76 (52.8%)	0.768
	Lesion Locations	16 (61.5%)	35 (79.5%)	52 (70.3%)	103 (71.5%)	0.257
	Total (0–5)	3.2 ± 1.4	3.1 ± 1.3	3.1 ± 1.4	3.1 ± 1.3	0.851
Factual Knowledge: Transmission	Transmission Pathways	25 (96.2%)	40 (90.9%)	68 (91.9%)	133 (92.4%)	0.839
	Vertical Transmission	14 (53.8%)	18 (40.9%)	20 (27%)	52 (36.1%)	0.096
	Sexual Transmission	19 (73.1%)	25 (56.8%)	51 (68.9%)	95 (66%)	0.285
	Total (0–4)	2.9 ± 1.0	2.6 ± 1.2	2.4 ± 1.0	2.5 ± 1.1	0.179
Factual Knowledge: Prevention	Vaccine Availability	5 (19.2%)	9 (20.5%)	20 (27%)	34 (23.6%)	0.607
	Pre-exposure Prophylaxis	20 (76.9%)	31 (70.5%)	56 (75.7%)	107 (74.3%)	0.776
	Cross-immunisation	19 (73.1%)	28 (63.6%)	55 (74.3%)	102 (70.8%)	0.449
	Total (0–4)	2.0 ± 1.2	2.1 ± 1.2	2.2 ± 1.2	2.1 ± 1.2	0.661
Factual Knowledge: Treatment	Treatment Availability	4 (15.4%)	9 (20.5%)	15 (20.3%)	28 (19.4%)	0.846
	Medications List	4 (15.4%)	5 (11.4%)	16 (21.6%)	25 (17.4%)	0.391
	Prognosis	8 (30.8%)	17 (38.6%)	30 (40.5%)	55 (38.2%)	0.676
	Total (0–4)	0.7 ± 0.7	0.7 ± 0.8	0.9 ± 1.0	0.8 ± 0.9	0.722

The Chi-squared (*χ^2^*) test, Fisher’s exact test, and Kruskal–Wallis (*H*) test were used with a significance level (*p*.) of ≤0.05.

**Table 5:** Correlation between Perceived and Factual Knowledge of Belarusian Healthcare Professionals Participating in the mpox Survey, October 2022, (*n* = 144).

			Perceived Knowledge				
			Epidemiology	Clinical Presentation	Transmission	Prevention	Treatment
Factual Knowledge	Epidemiology	*rho*	0.428	0.400	0.413	0.342	0.356
		*p.*	<0.001	<0.001	<0.001	<0.001	<0.001
	Clinical Presentation	*rho*	0.309	0.344	0.351	0.184	0.181
		*p.*	<0.001	<0.001	<0.001	<0.001	<0.001
	Transmission	*rho*	0.274	0.342	0.333	0.272	0.205
		*p.*	<0.001	<0.001	<0.001	<0.001	<0.001
	Prevention	*rho*	0.280	0.298	0.344	0.393	0.287
		*p.*	<0.001	<0.001	<0.001	<0.001	<0.001
	Treatment	*rho*	0.239	0.260	0.219	0.325	0.282
		*p.*	<0.001	<0.001	<0.001	<0.001	<0.001
	Overall Score	*rho*	0.439	0.481	0.494	0.426	0.380
		*p.*	<0.001	<0.001	<0.001	<0.001	<0.001

Non-parametric correlation (Spearman’s rho) was used with a significance level (*p*.) of ≤0.05.

**Table 6:** mpox Vaccine-related Perceptions of Belarusian Healthcare Professionals Participating in the mpox Survey, October 2022, (*n* = 144).

Category	Item	Mpox VAX Rejection (*n* = 26)	Mpox VAX Hesitancy (*n* = 44)	Mpox VAX Acceptance (*n* = 74)	Total (*n* = 144)	*p.*
Perceived Susceptibility	1. due to my occupation.	2.4 ± 1.2	2.8 ± 1.0	3.1 ± 1.1	2.9 ± 1.1	0.016
	2. due to lifestyle and health status.	2.2 ± 1.0	2.3 ± 1.0	2.2 ± 0.9	2.2 ± 0.9	0.673
	3. not vaccinated vs. smallpox.	2.5 ± 0.9	2.9 ± 1.0	3.0 ± 1.2	2.9 ± 1.1	0.148
	Overall Score (3–15)	7.1 ± 2.6	8.0 ± 2.3	8.3 ± 2.5	8.0 ± 2.5	0.126
Perceived Severity	1. I will be very sick.	2.5 ± 1.0	2.9 ± 0.9	2.8 ± 0.8	2.8 ± 0.9	0.137
	2. I may require hospitalisation.	3.0 ± 1.2	3.2 ± 0.9	3.3 ± 0.9	3.2 ± 0.9	0.357
	3. I might die.	2.7 ± 1.1	3.2 ± 0.9	3.0 ± 0.9	3.0 ± 1.0	0.047
	Overall Score (3–15)	8.2 ± 2.8	9.3 ± 2.4	9.0 ± 2.0	8.9 ± 2.3	0.117
Perceived Benefits	1. protected from getting infected.	2.4 ± 1.1	3.3 ± 0.8	3.3 ± 1.2	3.2 ± 1.1	<0.001
	2. protected from serious complications.	2.9 ± 1.2	3.7 ± 0.8	4.2 ± 0.7	3.8 ± 1.0	<0.001
	3. protect my patients and family.	2.7 ± 1.1	3.4 ± 0.9	4.0 ± 0.8	3.6 ± 1.0	<0.001
	Overall Score (3–15)	8.0 ± 3.1	10.5 ± 2.2	11.5 ± 2.0	10.6 ± 2.6	<0.001
Perceived Barriers	1. availability of mpox vaccine.	2.6 ± 1.1	3.1 ± 1.0	3.8 ± 1.1	3.4 ± 1.2	<0.001
	2. safety of mpox vaccine.	3.0 ± 1.2	3.2 ± 1.0	3.2 ± 1.1	3.2 ± 1.1	0.768
	3. effectiveness of mpox vaccine.	3.3 ± 1.2	3.2 ± 0.9	3.0 ± 1.1	3.1 ± 1.1	0.542
	Overall Score (3–15)	8.9 ± 2.4	9.5 ± 2.3	10.0 ± 2.5	9.6 ± 2.4	0.134
Cues to Action	1. mandated by the employer.	2.3 ± 1.1	2.8 ± 1.0	2.9 ± 1.2	2.8 ± 1.1	0.079
	2. recommended by health authorities.	2.6 ± 1.1	3.4 ± 0.8	3.5 ± 1.1	3.3 ± 1.1	<0.001
	3. reliable evidence on eff. and safety.	3.5 ± 1.3	4.0 ± 0.7	4.4 ± 0.9	4.1 ± 1.0	<0.001
	Overall Score (3–15)	8.4 ± 2.9	10.3 ± 1.6	10.8 ± 2.3	10.2 ± 2.4	<0.001

The Kruskal–Wallis (*H*) test was used with a significance level (*p*.) of ≤0.05.

**Table 7:** Correlation between mpox Vaccine Perceptions and Acceptance of Belarusian Healthcare Professionals Participating in the mpox Survey, October 2022, (*n* = 144).

		Perceived Susceptibility	Perceived Severity	Perceived Benefits	Perceived Barriers	Cues to Action	Acceptance
Perceived Susceptibility	*rho*	1.000	0.222	0.112	0.112	−0.004	0.111
	*p.*		0.007	0.180	0.181	0.967	0.185
Perceived Severity	*rho*	0.222	1.000	0.169	0.189	0.092	0.059
	*p.*	0.007		0.042	0.023	0.270	0.481
Perceived Benefits	*rho*	0.112	0.169	1.000	0.171	0.456	0.451
	*p.*	0.180	0.042		0.041	<0.001	<0.001
Perceived Barriers	*rho*	0.112	0.189	0.171	1.000	0.072	0.102
	*p.*	0.181	0.023	0.041		0.390	0.224
Cues to Action	*rho*	−0.004	0.092	0.456	0.072	1.000	0.349
	*p.*	0.967	0.270	<0.001	0.390		<0.001
Acceptance	*rho*	0.111	0.059	0.451	0.102	0.349	1.000
	*p.*	0.185	0.481	<0.001	0.224	<0.001	

Non-parametric correlation (Spearman’s rho) was used with a significance level (*p*.) of ≤0.05.

**Table 8:** mpox Vaccine Recommendation and Willingness to Pay (WTP) of Belarusian Healthcare Professionals Participating in the mpox Survey, October 2022, (*n* = 144).

Variable	Outcome	Mpox VAX Rejection (*n* = 26)	Mpox VAX Hesitancy (*n* = 44)	Mpox VAX Acceptance (*n* = 74)	Total (*n* = 144)	*p.*
I am willing/interested in recommending mpox vaccination to my patients, family members, and friends, especially those at risk.	Strongly disagree	7 (26.9%)	1 (2.3%)	0 (0%)	8 (5.6%)	<0.001
	Disagree	10 (38.5%)	0 (0%)	0 (0%)	10 (6.9%)	<0.001
	Not sure	5 (19.2%)	30 (68.2%)	4 (5.4%)	39 (27.1%)	<0.001
	Agree	3 (11.5%)	12 (27.3%)	48 (64.9%)	63 (43.8%)	<0.001
	Strongly agree	1 (3.8%)	1 (2.3%)	22 (29.7%)	24 (16.7%)	<0.001
	Total (µ ± SD)	2.3 ± 1.1	3.3 ± 0.6	4.2 ± 0.5	3.6 ± 1.0	<0.001
How much would you like to pay for the mpox vaccine shot as a personal expense?	It should be free.	15 (57.7%)	13 (29.5%)	3 (4.1%)	31 (21.5%)	0.001
	<BYN 24.7/dose	3 (11.5%)	14 (31.8%)	24 (32.4%)	41 (28.5%)	0.107
	BYN 24.71–121.10/dose	8 (30.8%)	16 (36.4%)	44 (59.5%)	68 (47.2%)	0.009
	BYN 121.11–244.67/dose	0 (0%)	1 (2.3%)	2 (2.7%)	3 (2.1%)	1.000
	≥BYN 244.68/dose	0 (0%)	0 (0%)	1 (1.4%)	1 (0.7%)	1.000
What is the optimal price of the mpox vaccine for the public?	It should be free.	14 (53.8%)	14 (31.8%)	23 (31.1%)	51 (35.4%)	0.095
	<BYN 24.7/dose	8 (30.8%)	19 (43.2%)	30 (40.5%)	57 (39.6%)	0.574
	BYN 24.71–121.10/dose	4 (15.4%)	11 (25%)	21 (28.4%)	36 (25%)	0.421

The Chi-squared (*χ^2^*) test, Fisher’s exact test, and Kruskal–Wallis (*H*) test were used with a significance level (*p*.) of ≤0.05.

## Data Availability

The data that support the findings of this study are available from the corresponding author upon reasonable request.

## Supplementary Materials

The following supporting information can be downloaded at: https://www.mdpi.com/article/10.3390/vaccines11081368/s1. Table S1: Predictors of mpox-related Perceived Knowledge of Belarusian Healthcare Workers Responding to mpox Survey; Table S2: Factual Knowledge of Belarusian Healthcare Workers Responding to mpox Survey; Table S3: Predictors of mpox-related Factual Knowledge of Belarusian Healthcare Workers Responding to mpox Survey. Table S4: Predictors of mpox vaccine-related Perceptions and Acceptance of Belarusian Healthcare Workers Responding to mpox Survey.
