# Supplementary material for: Belarusian Healthcare Professionals’ Views on Monkeypox and Vaccine Hesitancy
Source: Vaccines (Basel). 2023 Aug 15;11(8):1368. doi: 10.3390/vaccines11081368 (PMC10459544; doi:10.3390/vaccines11081368)
Supplement: Supplementary file 1 [file vaccines-11-01368-s001.zip › vaccines-2475104-supplementary.pdf]

Table S1. Predictors of MPOX-related Perceived Knowledge of Belarusian Healthcare Professionals Participating in the MPOX Survey, October 2022, (n = 144) 1

Variable	Outcome	Epidemiology	*p.*	Clinical Presentation	*p.*	Transmission	*p.*	Prevention	*p.*	Treatment	*p.*	Total	*p.*
Gender	Female	2.5 ± 0.9	0.039	2.8 ± 1.0	0.127	3.0 ± 1.0	0.328	2.5 ± 0.9	0.498	2.3 ± 0.9	0.133	13.1 ± 4.0	0.130
	Male	3.1 ± 1.0		3.3 ± 1.0		3.3 ± 1.0		2.8 ± 1.0		2.9 ± 1.1		15.3 ± 4.7	
Sexual Orientation	Heterosexual	2.6 ± 0.9	0.910	2.8 ± 1.0	0.807	3.0 ± 1.0	0.477	2.6 ± 0.9	**0.045**	2.4 ± 0.9	0.103	13.2 ± 4.0	0.388
	Homosexual	3.0 ± 0.0		3.0 ± 0.0		4.0 ± 0.0		3.0 ± 0.0		4.0 ± 0.0		17.0 ± 0.0	
	Bisexual	2.7 ± 0.8		3.2 ± 0.4		3.3 ± 0.5		2.8 ± 0.4		2.8 ± 0.8		14.8 ± 1.6	
	Other	2.5 ± 1.0		1.9 ± 2.8		2.5 ± 1.9		1.3 ± 0.5		1.8 ± 1.0		10.5 ± 5.5	
Age Group	≤ 31 years-old	2.5 ± 0.9	0.423	2.7 ± 0.9	0.231	2.9 ± 1.0	0.293	2.6 ± 1.0	0.156	2.4 ± 0.9	0.937	13.1 ± 4.2	0.998
	> 31 years-old	2.6 ± 0.8		2.9 ± 1.0		3.1 ± 1.1		2.4 ± 0.9		2.4 ± 0.9		13.4 ± 3.9	
Marital Status	Single	2.7 ± 0.8	0.654	3.2 ± 0.9	0.119	3.1 ± 0.9	0.607	2.8 ± 0.9	0.397	2.8 ± 0.9	0.061	14.5 ± 3.5	0.332
	Married	2.5 ± 0.9		2.7 ± 1.0		2.9 ± 1.1		2.5 ± 1.0		2.3 ± 0.9		13.0 ± 4.1	
	Widowed	2.0 ± 0.0		4.0 ± 0.0		4.0 ± 0.0		2.0 ± 0.0		1.0 ± 0.0		13.0 ± 0.0	
	Divorced	2.5 ± 0.8		3.0 ± 1.1		3.0 ± 1.1		2.5 ± 1.2		2.2 ± 1.0		13.2 ± 4.2	
Having Minors	No	2.6 ± 0.9	0.442	3.0 ± 1.0	0.129	3.1 ± 1.0	0.212	2.6 ± 1.0	0.311	2.5 ± 1.0	0.421	13.8 ± 4.2	0.090
	Yes	2.5 ± 0.8		2.7 ± 0.9		2.9 ± 1.0		2.5 ± 0.9		2.3 ± 0.8		12.9 ± 3.9	
Profession	Medical HCPs	2.6 ± 0.9	0.958	2.9 ± 1.0	0.542	3.0 ± 1.0	0.893	2.6 ± 0.9	0.479	2.4 ± 0.9	0.732	13.3 ± 4.1	0.473
	Allied HCPs	2.6 ± 0.8		2.8 ± 0.9		3.0 ± 1.1		2.4 ± 1.0		2.3 ± 0.9		13.0 ± 3.9	
Providing Care	No	2.5 ± 0.9	0.277	2.8 ± 1.0	0.806	3.0 ± 1.1	0.691	2.5 ± 1.0	0.188	2.3 ± 0.9	0.086	13.1 ± 4.1	0.352
	Yes	2.7 ± 0.7		2.9 ± 0.9		2.9 ± 0.9		2.7 ± 0.8		2.6 ± 0.9		13.8 ± 3.7	
Chronic Illnesses	No	2.6 ± 0.9	0.916	2.8 ± 1.0	0.653	2.8 ± 1.0	0.065	2.6 ± 1.0	0.820	2.4 ± 0.9	0.385	13.1 ± 4.3	0.776
	Yes	2.6 ± 0.8		2.9 ± 0.9		3.2 ± 1.0		2.5 ± 0.9		2.3 ± 0.9		13.4 ± 3.7	
Medical Treatments	No	2.6 ± 0.9	0.411	2.9 ± 1.0	0.480	3.0 ± 1.0	0.645	2.6 ± 1.0	0.134	2.5 ± 0.9	**0.005**	13.5 ± 4.2	0.053
	Yes	2.5 ± 0.8		2.8 ± 0.9		2.9 ± 1.0		2.4 ± 0.8		2.0 ± 0.8		12.5 ± 3.4	
COVID-19 Vaccination	No	2.4 ± 1.0	0.203	2.5 ± 1.1	0.095	2.7 ± 1.2	0.147	2.3 ± 1.1	0.208	2.1 ± 0.9	**0.041**	12.0 ± 4.5	0.065
	Yes	2.6 ± 0.8		2.9 ± 0.9		3.0 ± 1.0		2.6 ± 0.9		2.4 ± 0.9		13.6 ± 3.9	
COVID-19 Vaccine Doses	One Dose	2.3 ± 1.0	0.436	2.8 ± 1.3	0.663	2.8 ± 1.3	0.848	2.5 ± 1.0	0.506	1.5 ± 1.0	0.301	11.8 ± 5.0	0.697
	Two Doses	2.5 ± 0.8		2.8 ± 0.9		2.9 ± 1.0		2.6 ± 0.9		2.5 ± 0.9		13.2 ± 3.9	
	Three Doses	2.7 ± 0.9		3.0 ± 1.0		3.1 ± 1.0		2.6 ± 0.9		2.6 ± 0.9		13.9 ± 3.9	
	Four Doses	2.7 ± 0.8		3.0 ± 1.0		3.1 ± 1.1		2.5 ± 0.8		2.3 ± 0.8		13.6 ± 3.9	

	Five Doses or More	3.0 ± 0.8		3.3 ± 0.5		3.3 ± 0.5		3.5 ± 1.0		2.5 ± 1.0		15.5 ± 1.3	
Influenza Vaccination	No	2.1 ± 0.9	**0.003**	2.3 ± 1.1	**0.003**	2.5 ± 1.3	**0.011**	2.2 ± 1.0	**0.022**	2.2 ± 1.0	0.232	11.2 ± 4.8	**0.010**
	Yes	2.7 ± 0.8		3.0 ± 0.9		3.1 ± 0.9		2.6 ± 0.9		2.4 ± 0.9		13.7 ± 3.7	
Last 12 Months	No	2.6 ± 0.8	0.612	3.0 ± 0.8	0.289	3.2 ± 0.9	0.368	2.8 ± 0.9	0.180	2.5 ± 0.9	0.675	14.0 ± 3.4	0.444
	Yes	2.7 ± 0.9		2.9 ± 0.9		3.0 ± 1.0		2.5 ± 1.0		2.4 ± 0.9		13.5 ± 3.9	
Smallpox Vaccine	No	2.6 ± 0.8	0.768	2.8 ± 0.9	0.855	3.0 ± 1.0	0.722	2.6 ± 0.9	0.217	2.4 ± 0.9	0.472	13.3 ± 4.0	0.650
	Yes	2.6 ± 1.0		2.8 ± 1.1		3.0 ± 1.1		2.3 ± 1.0		2.3 ± 0.9		13.0 ± 4.4	
Undergrad Curriculum	No	2.5 ± 0.9	**0.011**	2.7 ± 0.9	**0.012**	2.9 ± 1.0	**0.005**	2.5 ± 1.0	0.147	2.3 ± 0.9	**0.023**	12.8 ± 4.0	**0.001**
	Yes	2.9 ± 0.8		3.2 ± 1.0		3.4 ± 1.0		2.7 ± 0.9		2.7 ± 0.9		15.0 ± 3.7	
Utilized Sources	Ministry of Health	2.7 ± 0.9	0.095	3.0 ± 1.0	0.066	3.1 ± 1.0	0.093	2.6 ± 0.9	0.438	2.5 ± 0.9	0.093	13.9 ± 3.9	0.061
	Public Health Institutes	2.9 ± 0.9	**0.012**	3.2 ± 0.8	**0.012**	3.4 ± 0.9	**0.013**	2.8 ± 0.9	0.128	2.6 ± 0.8	0.058	14.9 ± 3.2	**0.013**
	ECDC	3.0 ± 0.7	**0.004**	3.3 ± 0.7	**0.021**	3.5 ± 0.7	**0.013**	3.0 ± 0.6	**0.003**	2.8 ± 0.8	**0.005**	15.6 ± 2.5	**0.002**
	US CDC	3.0 ± 0.8	**0.008**	3.2 ± 0.6	**0.044**	3.4 ± 0.9	**0.027**	3.1 ± 0.8	**0.002**	2.8 ± 1.0	**0.010**	15.6 ± 3.0	**0.002**
	WHO	2.8 ± 0.7	**0.002**	3.1 ± 0.8	**<0.001**	3.3 ± 0.8	**<0.001**	2.8 ± 0.7	**<0.001**	2.6 ± 0.8	**0.003**	14.5 ± 3.0	**<0.001**
	Pharmaceutical Industry	2.3 ± 0.7	0.225	2.9 ± 0.9	0.957	2.8 ± 0.9	0.495	2.6 ± 0.8	0.846	2.4 ± 0.7	0.937	13.0 ± 3.4	0.539
	Professional Associations	2.9 ± 0.8	**0.009**	3.1 ± 0.8	0.065	3.2 ± 0.9	0.063	2.8 ± 0.9	0.128	2.5 ± 0.8	0.150	14.5 ± 3.4	**0.043**
	Scientific Journals	2.9 ± 0.8	**0.026**	3.0 ± 0.8	0.302	3.2 ± 1.0	0.120	2.9 ± 1.0	**0.008**	2.8 ± 1.0	**0.008**	14.8 ± 3.9	**0.006**
	Social Media	2.4 ± 0.9	**0.014**	2.7 ± 0.9	0.068	2.9 ± 1.0	0.239	2.5 ± 1.0	0.654	2.3 ± 0.9	0.591	12.9 ± 4.0	0.068
	News Portals	2.7 ± 0.8	0.097	3.0 ± 0.8	0.248	3.2 ± 0.9	0.058	2.8 ± 0.9	**0.030**	2.6 ± 0.8	**0.025**	14.3 ± 3.3	**0.039**
Overall Score: μ ± SD (min-max)		2.6 ± 0.9 (1 – 5)		2.8 ± 1.0 (1 – 5)		3.0 ± 1.0 (1 – 5)		2.5 ± 0.9 (1 – 5)		2.4 ± 0.9 (1 – 5)		13.3 ± 4.0 (5 – 25)	

Kruskal-Wallis (*H*) test and Mann-Whitney (*U*) test were used with a significance level (*p.*) of ≤ 0.05.

Table S2. Factual Knowledge of Belarusian Healthcare Professionals Participating in the MPOX Survey, October 2022, (*n* = 144)

	**Question**	**Answer**	**MPOX VAX Rejection (*n* = 26)**	**MPOX VAX Hesitancy (*n* = 44)**	**MPOX VAX Acceptance (*n* = 74)**	**Total (*n* = 144)**	***p.***
**Epidemiology**	The incubation period of MPOX ranges between …	3 – 7 days	6 (23.1%)	6 (13.6%)	10 (13.5%)	22 (15.3%)	0.454
		**7 – 21 days**	13 (50%)	26 (59.1%)	46 (62.2%)	85 (59%)	0.555
		21 – 28 days	1 (3.8%)	6 (13.6%)	5 (6.8%)	12 (8.3%)	0.364
		I do not know	6 (23.1%)	6 (13.6%)	13 (17.6%)	25 (17.4%)	0.558
	The case-fatality ratio of MPOX usually ranges between …	**4% and 11%**	13 (50%)	23 (52.3%)	50 (67.6%)	86 (59.7%)	0.140
		14% and 19%	2 (7.7%)	5 (11.4%)	6 (8.1%)	13 (9%)	0.925
		20% and 30%	6 (23.1%)	4 (9.1%)	4 (5.4%)	14 (9.7%)	**0.039**

		I do not know	5 (19.2%)	12 (27.3%)	14 (18.9%)	31 (21.5%)	0.538
	MPOX used to be endemic in …	Southeast Asia	1 (3.8%)	2 (4.5%)	6 (8.1%)	9 (6.3%)	0.728
		**Sub-Saharan Africa**	20 (76.9%)	30 (68.2%)	52 (70.3%)	102 (70.8%)	0.731
		North America	0 (0%)	2 (4.5%)	1 (1.4%)	3 (2.1%)	0.438
		I do not know	5 (19.2%)	10 (22.7%)	15 (20.3%)	30 (20.8%)	0.928
**Clinical Presentation**	Which of the following are symptoms of MPOX infection? (*select all that apply*)	Fever	22 (84.6%)	38 (86.4%)	63 (85.1%)	123 (85.4%)	1.000
		Chills	20 (76.9%)	17 (38.6%)	39 (52.7%)	76 (52.8%)	**0.008**
		Headache	16 (61.5%)	18 (40.9%)	38 (51.4%)	72 (50%)	0.235
		Myalgia	13 (50%)	18 (40.9%)	36 (48.6%)	67 (46.5%)	0.664
		Lymphadenopathy	19 (73.1%)	27 (61.4%)	51 (68.9%)	97 (67.4%)	0.552
		Fatigue	16 (61.5%)	22 (50%)	36 (48.6%)	74 (51.4%)	0.515
		Respiratory symptoms	10 (38.5%)	14 (31.8%)	26 (35.1%)	50 (34.7%)	0.848
		Skin and mucosal lesions	24 (92.3%)	37 (84.1%)	62 (83.8%)	123 (85.4%)	0.569
		I do not know	2 (7.7%)	5 (11.4%)	9 (12.2%)	16 (11.1%)	0.938
	… is/are the diagnostic feature(s) of MPOX that distinguish(es) it from smallpox.	**Lymphadenopathy**	14 (53.8%)	25 (56.8%)	37 (50%)	76 (52.8%)	0.768
		Fever	0 (0%)	4 (9.1%)	1 (1.4%)	5 (3.5%)	0.081
		Respiratory symptoms	7 (26.9%)	4 (9.1%)	15 (20.3%)	26 (18.1%)	0.119
		I do not know	5 (19.2%)	11 (25%)	21 (28.4%)	37 (25.7%)	0.651
	The possible locations to find MPOX lesions are … (*select all that apply*)	Genitalia	13 (50%)	16 (36.4%)	37 (50%)	66 (45.8%)	0.318
		Anus	10 (38.5%)	10 (22.7%)	24 (32.4%)	44 (30.6%)	0.340
		Extremities	13 (50%)	20 (45.5%)	35 (47.3%)	68 (47.2%)	0.934
		Chest	10 (38.5%)	19 (43.2%)	30 (40.5%)	59 (41%)	0.922
		Back	10 (38.5%)	19 (43.2%)	26 (35.1%)	55 (38.2%)	0.685
		Face and Mouth	13 (50%)	26 (59.1%)	41 (55.4%)	80 (55.6%)	0.760
		I do not know	10 (38.5%)	11 (25%)	23 (31.1%)	44 (30.6%)	0.493
**Transmission**	MPOX can be transmitted through … (*select all that apply*)	contact with respiratory secretions.	13 (50%)	28 (63.6%)	40 (54.1%)	81 (56.3%)	0.465
		touching objects and fabrics used by someone with MPOX.	11 (42.3%)	16 (36.4%)	28 (37.8%)	55 (38.2%)	0.881
		direct contact with MPOX rash, scabs or body fluids.	22 (84.6%)	33 (75%)	61 (82.4%)	116 (80.6%)	0.520
		I do not know	1 (3.8%)	4 (9.1%)	7 (9.5%)	12 (8.3%)	0.785
	Vertical transmission, from a pregnant person to their fetus through placenta, of MPOX is possible.	**True**	14 (53.8%)	18 (40.9%)	20 (27%)	52 (36.1%)	**0.036**
		False	4 (15.4%)	11 (25%)	16 (21.6%)	31 (21.5%)	0.639

		I do not know	8 (30.8%)	15 (34.1%)	38 (51.4%)	61 (42.4%)	0.078
	Sexual transmission of MPOX is possible among …	homosexual partners only.	3 (11.5%)	6 (13.6%)	6 (8.1%)	15 (10.4%)	0.623
		**both of homo- and heterosexual partners.**	19 (73.1%)	25 (56.8%)	51 (68.9%)	95 (66%)	0.285
		I do not know	4 (15.4%)	13 (29.5%)	17 (23%)	34 (23.6%)	0.396
**Prevention**	An effective vaccine against MPOX is to date available.	**Ture**	5 (19.2%)	9 (20.5%)	20 (27%)	34 (23.6%)	0.607
		False	11 (42.3%)	21 (47.7%)	31 (41.9%)	63 (43.8%)	0.815
		I do not know	10 (38.5%)	14 (31.8%)	23 (31.1%)	47 (32.6%)	0.780
	Pre-exposure Prophylaxis (PrEP) of MPOX … *(select all that apply)*	should be administered to healthcare professionals caring for MPOX patients.	14 (53.8%)	29 (65.9%)	48 (64.9%)	91 (63.2%)	0.547
		should be administered to laboratory workers dealing with *orthopoxviruses* samples and cultures.	10 (38.5%)	22 (50%)	34 (45.9%)	66 (45.8%)	0.645
		should be administered to individuals with contact/exposure to MPOX.	13 (50%)	24 (54.5%)	49 (66.2%)	86 (59.7%)	0.245
		can prevent the onset of the disease if administered until four days after exposure.	9 (34.6%)	8 (18.2%)	22 (29.7%)	39 (27.1%)	0.250
		I do not know	6 (23.1%)	13 (29.5%)	18 (24.3%)	37 (25.7%)	0.776
	Which vaccine(s) provide(s) cross-immunization against MPOX?	Chickenpox Vaccine	2 (7.7%)	2 (4.5%)	6 (8.1%)	10 (6.9%)	0.827
		**Smallpox Vaccine**	19 (73.1%)	28 (63.6%)	55 (74.3%)	102 (70.8%)	0.449
		COVID-19 Vaccine	0 (0%)	0 (0%)	1 (1.4%)	1 (0.7%)	1.000
		None of the above	2 (7.7%)	5 (11.4%)	6 (8.1%)	13 (9%)	0.925
		I do not know	3 (11.5%)	9 (20.5%)	6 (8.1%)	18 (12.5%)	0.154
**Treatment**	Effective drugs against MPOX are currently available.	**Ture**	4 (15.4%)	9 (20.5%)	15 (20.3%)	28 (19.4%)	0.846
		False	13 (50%)	19 (43.2%)	37 (50%)	69 (47.9%)	0.752
		I do not know	9 (34.6%)	16 (36.4%)	22 (29.7%)	47 (32.6%)	0.738
	For severe MPOX cases, … can be used. *(select all that apply)*	tecovirimat	3 (11.5%)	4 (9.1%)	10 (13.5%)	17 (11.8%)	0.886
		brincidofovir	1 (3.8%)	2 (4.5%)	3 (4.1%)	6 (4.2%)	1.000
		cidofovir	2 (7.7%)	0 (0%)	9 (12.2%)	11 (7.6%)	0.031
		I do not know	22 (84.6%)	39 (88.6%)	59 (79.7%)	120 (83.3%)	0.499
	For most cases, MPOX infection leads to …	**mild and self-limiting clinical course.**	8 (30.8%)	17 (38.6%)	30 (40.5%)	55 (38.2%)	0.676
		moderate yet life-threatening clinical course.	11 (42.3%)	19 (43.2%)	32 (43.2%)	62 (43.1%)	0.996
		fatal and life-threatening clinical course.	6 (23.1%)	4 (9.1%)	3 (4.1%)	13 (9%)	0.022
		I do not know	1 (3.8%)	4 (9.1%)	9 (12.2%)	14 (9.7%)	0.563

Chi-squared (χ^2) test and Fisher's exact test were used with a significance level (*p*.) of ≤ 0.05.

Table S3. Predictors of MPOX-related Factual Knowledge of Belarusian Healthcare Professionals Participating in the MPOX Survey, October 2022, (*n* = 144)

**Variable**	**Outcome**	**Epidemiology**	***p.***	**Clinical Presentation**	***p.***	**Transmission**	***p.***	**Prevention**	***p.***	**Treatment**	***p.***	**Total**	***p.***
Gender	Female	1.9 ± 1.0	0.175	3.1 ± 1.4	0.648	2.5 ± 1.1	0.058	2.1 ± 1.2	0.530	0.8 ± 0.9	0.134	10.3 ± 4.0	0.105
	Male	2.4 ± 0.7		3.4 ± 0.9		3.3 ± 0.9		2.4 ± 1.2		1.3 ± 1.0		12.6 ± 3.2	
Sexual Orientation	Heterosexual	1.9 ± 1.0	0.593	3.1 ± 1.4	0.492	2.5 ± 1.1	0.324	2.1 ± 1.2	0.469	0.8 ± 0.9	0.652	10.5 ± 3.9	0.267
	Homosexual	1.0 ± 0.0		2.0 ± 0.0		2.0 ± 0.0		1.0 ± 0.0		0.0 ± 0.0		6.0 ± 0.0	
	Bisexual	2.0 ± 0.9		3.2 ± 1.8		3.2 ± 0.8		2.7 ± 1.5		1.0 ± 1.1		12.0 ± 5.0	
	Other	1.5 ± 1.0		2.5 ± 1.0		2.0 ± 1.2		2.0 ± 0.8		0.5 ± 0.6		8.5 ± 3.8	
Age Group	≤ 31 years-old	1.8 ± 1.0	0.253	3.1 ± 1.3	0.972	2.6 ± 0.9	0.617	2.2 ± 1.2	0.232	0.8 ± 0.9	0.743	10.5 ± 3.6	0.774
	> 31 years-old	2.0 ± 1.0		3.1 ± 1.4		2.5 ± 1.2		2.0 ± 1.2		0.8 ± 1.0		10.4 ± 4.3	
Marital Status	Single	1.9 ± 1.1	0.441	3.1 ± 1.1	0.747	2.6 ± 1.1	0.110	2.7 ± 1.0	**0.046**	0.8 ± 0.8	0.297	11.2 ± 3.3	0.753
	Married	1.9 ± 1.0		3.1 ± 1.4		2.5 ± 1.1		2.0 ± 1.2		0.8 ± 0.9		10.2 ± 4.2	
	Widowed	3.0 ± 0.0		4.0 ± 0.0		3.0 ± 0.0		2.0 ± 0.0		0.0 ± 0.0		12.0 ± 0.0	
	Divorced	1.5 ± 0.9		3.5 ± 1.0		3.5 ± 0.5		2.3 ± 1.0		0.3 ± 0.8		11.2 ± 2.0	
Having Minors	No	1.9 ± 1.1	0.641	3.2 ± 1.1	0.700	2.6 ± 1.0	0.639	2.4 ± 1.2	**0.012**	0.7 ± 0.8	0.428	10.9 ± 3.3	0.349
	Yes	1.9 ± 1.0		3.0 ± 1.5		2.5 ± 1.1		2.0 ± 1.2		0.9 ±1.0		10.2 ± 4.3	
Profession	Medical HCPs	2.0 ± 1.0	0.090	3.2 ± 1.3	0.255	2.6 ± 1.1	0.265	2.2 ± 1.2	0.459	0.9 ± 0.9	0.140	10.8 ± 3.8	0.113
	Allied HCPs	1.7 ± 0.9		2.8 ± 1.5		2.3 ± 1.2		2.0 ± 1.3		0.6 ± 0.9		9.4 ± 4.3	
Providing Care	No	1.9 ± 1.0	0.788	3.0 ± 1.4	0.501	2.6 ±1.1	0.354	2.2 ± 1.3	0.744	0.9 ± 1.0	**0.010**	10.5 ± 4.1	0.536
	Yes	2.0 ± 0.9		3.3 ± 1.2		2.4 ± 1.0		2.1 ± 1.0		0.4 ± 0.6		10.2 ± 3.5	
Chronic Illnesses	No	1.8 ± 1.0	0.338	3.1 ± 1.4	0.674	2.4 ± 1.2	0.226	2.2 ± 1.2	0.338	0.8 ± 0.9	0.918	10.4 ± 4.2	0.932
	Yes	2.0 ± 0.9		3.1 ± 1.2		2.7 ± 1.0		2.1 ± 1.2		0.8 ± 0.9		10.6 ± 3.5	
Medical Treatments	No	1.9 ± 1.0	0.516	3.1 ± 1.3	0.379	2.5 ± 1.1	0.453	2.2 ± 1.2	0.674	0.8 ± 0.9	0.680	10.5 ± 4.0	0.780
	Yes	1.8 ± 1.0		2.9 ± 1.4		2.7 ± 1.0		2.1 ± 1.2		0.9 ± 0.9		10.3 ± 3.7	
COVID-19 Vaccination	No	1.9 ± 1.0	0.761	2.8 ± 1.4	0.237	2.4 ± 1.2	0.560	1.8 ± 1.2	0.102	0.8 ± 0.8	0.939	9.6 ± 3.8	0.235
	Yes	1.9 ± 1.0		3.2 ± 1.3		2.6 ± 1.1		2.2 ± 1.2		0.9 ± 1.0		10.7 ± 4.0	
COVID-19 Vaccine Doses	One Dose	1.5 ± 0.6	0.148	4.0 ± 0.8	0.536	2.5 ± 0.6	0.675	2.8 ± 1.0	0.326	0.0 ± 0.0	0.285	10.8 ± 1.3	0.863
	Two Doses	1.8 ± 1.0		3.0 ± 1.4		2.5 ± 1.2		2.1 ± 1.3		1.0 ± 1.1		10.4 ± 4.3	
	Three Doses	1.9 ± 1.1		3.3 ± 1.3		2.8 ± 1.0		2.4 ± 1.2		0.8 ± 0.9		11.1 ± 4.0	
	Four Doses	2.1 ± 1.0		3.1 ± 1.4		2.5 ± 1.1		1.8 ± 1.1		0.7 ± 0.7		10.3 ± 3.8	
	Five Doses or More	2.8 ± 0.5		2.8 ± 1.0		2.3 ± 0.5		2.5 ± 1.0		0.8 ± 0.5		11.0 ± 2.7	
Influenza	No	1.5 ± 1.0	**0.017**	2.3 ± 1.5	**0.002**	2.0 ± 1.3	**0.006**	1.7 ± 1.3	**0.035**	0.6 ± 0.8	0.240	8.0 ± 4.7	**0.004**

Vaccination	Yes	2.0 ± 1.0		3.3 ± 1.2		2.7 ± 1.0		2.3 ± 1.2		0.8 ± 0.9		11.0 ± 3.6	
Last 12 Months	No	2.0 ± 0.9	0.951	3.1 ± 1.3	0.230	2.7 ± 1.1	0.947	2.2 ± 1.1	0.421	0.9 ± 1.0	0.671	10.9 ± 3.5	0.657
	Yes	2.0 ± 1.0		3.4 ± 1.2		2.7 ± 1.0		2.3 ± 1.2		0.8 ± 0.9		11.2 ± 3.6	
Smallpox Vaccine	No	1.9 ± 1.0	0.519	3.1 ± 1.3	0.556	2.5 ± 1.0	0.344	2.2 ± 1.2	0.811	0.8 ± 1.0	0.567	10.6 ± 3.9	0.947
	Yes	1.8 ± 1.1		2.9 ± 1.6		2.6 ± 1.3		2.1 ± 1.2		0.7 ± 0.7		10.0 ± 4.1	
Undergrad Curriculum	No	1.8 ± 1.0	0.207	3.0 ± 1.4	0.541	2.5 ± 1.1	0.609	2.1 ± 1.2	0.111	0.7 ± 0.9	0.057	10.2 ± 4.0	0.127
	Yes	2.1 ± 1.0		3.3 ± 1.8		2.7 ± 1.0		2.5 ± 1.3		1.1 ± 1.0		11.6 ± 3.6	
Utilized Sources	Ministry of Health	1.9 ± 1.0	0.970	3.1 ± 1.4	0.680	2.6 ± 1.2	0.350	2.2 ± 1.1	0.611	0.8 ± 1.0	0.850	10.7 ± 4.1	0.359
	Public Health Institutes	1.9 ± 1.0	0.831	3.3 ± 1.3	0.407	2.8 ± 1.1	0.133	2.3 ± 1.1	0.298	0.8 ± 0.8	0.456	11.1 ± 3.9	0.328
	ECDC	2.3 ± 1.2	**0.022**	3.3 ± 1.1	0.395	3.1 ± 0.8	**0.008**	2.6 ± 1.0	**0.036**	0.8 ± 0.8	0.493	12.1 ± 3.8	**0.028**
	US CDC	2.5 ± 1.1	**<0.001**	3.8 ± 0.8	**0.004**	3.1 ± 0.8	**0.011**	3.0 ± 0.9	**<0.001**	0.9 ± 0.8	0.404	13.2 ± 3.0	**<0.001**
	WHO	2.1 ± 1.0	**0.004**	3.4 ± 1.2	**0.005**	2.8 ± 1.0	**0.006**	2.5 ± 1.1	**<0.001**	0.9 ± 1.1	0.315	11.6 ± 3.7	**<0.001**
	Pharmaceutical Industry	1.0 ± 0.9	**0.005**	3.4 ± 1.0	0.514	2.6 ± 1.0	0.980	2.1 ± 1.1	0.977	0.2 ± 0.4	**0.015**	9.3 ± 1.9	0.176
	Professional Associations	2.0 ± 1.0	0.314	3.4 ± 1.1	0.120	2.7 ± 0.9	0.633	2.2 ± 1.2	0.500	0.8 ± 0.9	0.949	11.1 ± 3.2	0.259
	Scientific Journals	1.9 ± 1.1	0.629	3.1 ± 1.1	0.775	2.7 ± 1.0	0.282	2.5 ± 1.2	0.074	0.8 ± 0.8	0.490	11.1 ± 4.1	0.299
	Social Media	1.8 ± 1.0	0.259	3.1 ± 1.3	0.528	2.6 ± 1.0	0.693	2.2 ± 1.3	0.337	0.8 ± 1.0	0.763	10.5 ± 3.9	0.878
	News Portals	2.0 ± 1.0	0.397	3.3 ± 1.2	0.271	2.7 ± 0.8	0.459	2.3 ± 1.2	0.326	0.8 ± 1.0	0.544	11.0 ± 3.3	0.361
Overall Score: μ ± SD (min-max)		1.9 ± 1.0 (0 – 3)		3.1 ± 1.3 (0 – 5)		2.5 ± 1.1 (0 – 4)		2.1 ± 1.2 (0 – 4)		0.8 ± 0.9 (0 – 4)		10.5 ± 3.9 (0 – 20)	

Kruskal-Wallis (*H*) test and Mann-Whitney (*U*) test were used with a significance level (*p.*) of ≤ 0.05.

Table S4. Predictors of MPOX Vaccine-related Perceptions and Acceptance of Belarusian Healthcare Professionals Participating in the MPOX Survey, October 2022, (*n* = 144) 7

Variable	Outcome	Perceived Susceptibility	*p.*	Perceived Severity	*p.*	Perceived Benefits	*p.*	Perceived Barriers	*p.*	Cues to Action	*p.*	Acceptance	*p.*
Gender	Female	8.1 ± 2.4	0.096	8.9 ± 2.3	0.215	10.5 ± 2.6	0.286	9.5 ± 2.4	0.155	10.1 ± 2.3	0.081	3.4 ± 1.1	0.841
	Male	6.5 ± 2.6		10.0 ± 2.1		11.4 ± 2.6		11.0 ± 2.6		11.4 ± 2.6		3.4 ± 1.1	
Sexual Orientation	Heterosexual	8.0 ± 2.5	0.749	9.0 ± 2.3	0.122	10.6 ± 2.5	0.931	9.7 ± 2.4	0.485	10.1 ± 2.3	0.551	3.4 ± 1.1	0.721
	Homosexual	9.0 ± 0.0		11.0 ± 0.0		10.0 ± 0.0		11.0 ± 0.0		9.0 ± 0.0		4.0 ± 0.0	
	Bisexual	8.3 ± 2.8		8.0 ± 1.3		10.3 ± 4.1		9.2 ± 2.6		10.5 ± 3.0		3.3 ± 1.5	
	Other	7.0 ± 2.2		6.7 ± 2.8		9.3 ± 4.2		7.8 ± 3.6		11.5 ± 3.3		4.0 ± 1.2	
Age Group	≤ 31 years-old	8.1 ± 2.3	0.582	8.7 ± 2.2	0.097	11.0 ± 2.4	0.052	9.8 ± 2.4	0.418	10.7 ± 2.1	**0.005**	3.6 ± 1.0	0.072
	> 31 years-old	7.9 ± 2.7		9.3 ± 2.4		10.1 ± 2.8		9.4 ± 2.5		9.6 ± 2.6		3.2 ± 1.1	
Marital Status	Single	8.1 ± 2.2	0.354	8.7 ± 1.9	**0.049**	10.2 ± 2.7	**0.031**	9.9 ± 2.2	0.246	10.6 ± 2.4	0.079	3.5 ± 1.3	**0.009**
	Married	8.0 ± 2.5		9.2 ± 2.3		10.8 ± 2.4		9.6 ± 2.5		10.2 ± 2.3		3.5 ± 1.0	
	Widowed	5.0 ± 0.0		8.0 ± 0.0		15.0 ± 0.0		11.0 ± 0.0		15.0 ± 0.0		4.0 ± 0.0	
	Divorced	6.7 ± 2.7		6.3 ± 2.5		7.3 ± 3.7		8.2 ± 1.3		8.3 ± 3.0		1.8 ± 0.8	
Having Minors	No	8.0 ± 2.5	0.960	8.8 ± 2.4	0.449	11.1 ± 2.6	**0.021**	9.8 ± 2.5	0.451	10.6 ± 2.4	0.143	3.6 ± 1.1	0.093
	Yes	8.0 ± 2.5		9.1 ± 2.3		10.2 ± 2.6		9.5 ± 2.4		9.9 ± 2.3		3.3 ± 1.1	
Profession	Medical HCPs	8.0 ± 2.5	0.683	8.8 ± 2.3	**0.040**	10.8 ± 2.6	**0.022**	9.7 ± 2.4	0.799	10.4 ± 2.3	**0.015**	3.5 ± 1.0	0.068
	Allied HCPs	7.8 ± 2.4		9.6 ± 2.3		10.0 ± 2.6		9.5 ± 2.7		9.3 ± 2.6		3.1 ± 1.2	
Providing Care	No	7.9 ± 2.5	0.229	9.0 ± 2.3	0.455	10.9 ± 2.3	**0.026**	9.7 ± 2.3	0.716	10.3 ± 2.2	0.159	3.4 ± 1.1	0.295
	Yes	8.3 ± 2.5		8.7 ± 2.1		9.5 ± 3.2		9.3 ± 2.8		9.7 ± 2.8		3.6 ± 1.1	
Chronic Illnesses	No	7.7 ± 2.7	0.064	8.8 ± 2.4	0.323	10.8 ± 2.7	0.235	9.7 ± 2.5	0.654	10.3 ± 2.4	0.304	3.5 ± 1.1	0.150
	Yes	8.4 ± 2.1		9.2 ± 2.1		10.3 ± 2.6		9.5 ± 2.4		10.0 ± 2.3		3.3 ± 1.0	
Medical Treatments	No	7.8 ± 2.4	0.174	8.7 ± 2.2	**0.046**	10.5 ± 2.5	0.296	9.8 ± 2.3	0.336	10.2 ± 2.4	0.795	3.4 ± 1.1	0.937
	Yes	8.6 ± 2.7		9.6 ± 2.4		11.0 ± 2.9		9.2 ± 2.9		10.3 ± 2.3		3.4 ± 1.0	
COVID-19 Vaccination	No	7.7 ± 2.2	0.405	8.8 ± 2.4	0.540	10.0 ± 2.9	0.050	9.7 ± 2.3	0.779	8.6 ± 2.4	**<0.001**	3.0 ± 1.2	0.057
	Yes	8.0 ± 2.5		9.0 ± 2.3		10.8 ± 2.5		9.6 ± 2.5		10.6 ± 2.2		3.5 ± 1.0	
COVID-19 Vaccine Doses	One Dose	7.8 ± 1.3	0.510	9.3 ± 2.5	0.776	10.5 ± 1.3	0.092	11.5 ± 1.7	0.332	10.3 ± 2.2	0.709	3.5 ± 1.3	0.885
	Two Doses	7.8 ± 2.0		8.8 ± 2.2		10.4 ± 2.2		9.5 ± 2.8		10.6 ± 1.7		3.5 ± 0.8	
	Three Doses	8.2 ± 2.6		9.0 ± 2.3		10.5 ± 3.0		9.4 ± 2.3		10.6 ± 2.5		3.5 ± 1.2	
	Four Doses	8.6 ± 3.4		9.6 ± 2.4		12.1 ± 1.9		9.9 ± 2.4		10.7 ± 2.6		3.6 ± 0.9	
	Five Doses or More	6.8 ± 2.2		8.3 ± 2.2		9.8 ± 3.3		9.5 ± 2.6		9.3 ± 2.2		3.8 ± 1.9	
Influenza Vaccination	No	7.9 ± 3.1	0.805	8.8 ± 3.2	0.814	9.6 ± 2.7	**0.007**	9.2 ± 2.8	0.367	8.3 ± 2.4	**<0.001**	2.9 ± 1.1	**0.003**
	Yes	8.0 ± 2.3		9.0 ± 2.1		10.8 ± 2.6		9.7 ± 2.4		10.6 ± 2.1		3.5 ± 1.0	

Last 12 Months	No	8.1 ± 2.0	0.847	9.3 ± 1.8	0.069	10.8 ± 2.1	0.470	9.8 ± 2.7	0.484	10.2 ± 2.1	0.108	3.5 ± 1.0	0.787
	Yes	7.9 ± 2.6		8.8 ± 2.2		10.8 ± 2.8		9.7 ± 2.1		10.9 ± 2.1		3.6 ± 1.1	
Smallpox Vaccine	No	8.1 ± 2.4	0.057	8.8 ± 2.2	0.124	10.7 ± 2.4	0.878	9.8 ± 2.3	0.096	10.3 ± 2.3	0.502	3.5 ± 1.0	0.104
	Yes	7.3 ± 2.6		9.7 ± 2.6		10.2 ± 3.5		8.7 ± 2.8		9.9 ± 2.6		3.1 ± 1.2	
Undergrad Curriculum	No	8.0 ± 2.5	0.591	8.9 ± 2.3	0.757	10.6 ± 2.6	0.984	9.6 ± 2.5	0.686	10.2 ± 2.4	0.545	3.5 ± 1.1	0.491
	Yes	7.7 ± 2.3		9.0 ± 2.2		10.5 ± 2.6		9.8 ± 2.3		10.1 ± 2.4		3.3 ± 1.1	
Utilized Sources	Ministry of Health	8.1 ± 2.2	0.770	9.0 ± 2.2	0.621	10.9 ± 2.5	0.220	9.7 ± 2.3	0.753	10.4 ± 2.2	0.576	3.5 ± 1.1	0.170
	Public Health Institutes	7.6 ± 2.4	0.411	8.4 ± 2.3	0.249	10.1 ± 3.1	0.633	9.9 ± 2.5	0.465	10.0 ± 2.1	0.282	3.3 ± 1.2	0.348
	ECDC	8.5 ± 3.0	0.160	9.0 ± 1.7	0.864	11.5 ± 1.7	0.053	10.5 ± 2.1	**0.045**	10.7 ± 1.4	0.343	3.5 ± 1.1	0.705
	US CDC	8.0 ± 2.7	0.857	8.9 ± 2.0	0.785	11.6 ± 1.7	**0.036**	9.8 ± 1.9	0.866	10.4 ± 2.2	0.439	3.6 ± 1.0	0.361
	WHO	7.9 ± 2.5	0.721	8.7 ± 2.0	0.154	10.4 ± 2.6	0.303	9.8 ± 2.4	0.308	10.1 ± 2.3	0.998	3.4 ± 1.1	0.872
	Pharmaceutical Industry	7.7 ± 1.9	0.564	9.6 ± 2.8	0.371	10.5 ± 2.1	0.513	10.9 ±1.7	0.063	10.6 ± 2.0	0.943	3.6 ± 1.0	0.706
	Professional Associations	7.3 ± 2.6	0.110	8.5 ± 2.5	0.119	9.9 ± 2.8	0.055	9.7 ± 2.0	0.912	10.2 ± 2.0	0.712	3.3 ± 1.2	0.554
	Scientific Journals	8.5 ± 2.5	0.124	8.7 ± 2.2	0.324	10.3 ± 3.0	0.855	9.9 ± 2.7	0.203	10.1 ± 2.2	0.942	3.5 ± 0.9	0.507
	Social Media	8.2 ± 2.4	0.160	9.1 ± 2.2	0.496	10.7 ± 2.3	0.798	9.7 ± 2.3	0.636	10.5 ± 2.0	**0.023**	3.6 ± 1.0	**0.021**
	News Portals	8.1 ± 2.5	0.528	8.9 ± 2.0	0.611	10.0 ± 2.6	0.066	9.9 ± 2.5	0.478	9.8 ± 2.3	0.200	3.5 ± 1.1	0.347
Overall Score: μ ± SD (min-max)		8.0 ± 2.5 (3 – 15)		8.9 ± 2.3 (3 – 15)		10.6 ± 2.6 (3 – 15)		9.6 ± 2.4 (2 – 10)		10.2 ± 2.4 (3 – 15)		3.4 ± 1.1 (1 – 5)	

Kruskal-Wallis (*H*) test and Mann-Whitney (*U*) test were used with a significance level (*p.*) of ≤ 0.05.
